# A Prospective Longitudinal Observational Study on the Emotional Impact of AI‐Simulated Smiles on Orthodontic Patient Motivation

**DOI:** 10.1155/ijod/8425551

**Published:** 2026-01-02

**Authors:** Thomas Mourgues, María José González-Olmo, Luis Huanca Ghislanzoni, Andrei Otel, Martín Romero-Maroto

**Affiliations:** ^1^ Department of Orthodontics, Rey Juan Carlos University, 28922, Alcorcón, Madrid, Spain, urjc.es; ^2^ Division of Orthodontics, Clinics of Dental Medicine, University of Geneva, 1205, Geneva, Switzerland, unige.ch; ^3^ Independent Researcher, Paris, France

**Keywords:** artificial intelligence in dentistry, digital smile design, esthetic perception, orthodontic motivation, patient decision-making, smile simulation, SmileView

## Abstract

**Background:**

To evaluate the impact of the SmileView (SV) simulation on smile esthetic perception and its influence on motivation to undertake orthodontic treatment.

**Methods:**

A longitudinal study was conducted with 51 subjects in Madrid, Spain. Participants had an initial smile photo taken (T0) and a simulated image generated by SV (T1). They rated, on a 1–10 scale, the general perception of their smile and specific variables (width, alignment, color, gingival exposure, and shape) at both T0 and T1. Intention to pursue orthodontic treatment was recorded. Data were analyzed with SPSS 28.0.

**Results:**

Smile perception improved significantly postsimulation (T0: 5.84 ± 2.12; T1: 7.00 ± 2.61; *p* = 0.038), with notable gains in alignment (*p*  < 0.001), color (*p* = 0.004), and shape (*p* = 0.008). No significant changes were found for width (*p* = 0.143) or gingival exposure (*p* = 0.721). Subjects with negative perception at T1 were influenced mainly by tooth shape (*p* = 0.033). Those with improved perception were driven by alignment, color, and shape. Intent to undergo orthodontic treatment decreased slightly (49.0%–45.0%). Influenced subjects were mostly women, aged 41, with higher education and socioeconomic status. While alignment, color, and shape influenced decision changes, perceived increase in smile width emerged as the strongest independent predictor of treatment intention.

**Conclusion:**

SV improves smile perception but has limited influence on treatment motivation without clinical guidance.

## 1. Introduction

Esthetics refers to the principles by which we perceive beauty. Orofacial esthetics focuses on the visual appearance of the face and smile, and has been associated with mental health issues and various aspects of social life [1]. Given that a decline in smile attractiveness is one of the main reasons individuals seek orthodontic treatment, the study of smile related self perception has become a fundamental area of scientific investigation in orthodontics [2–6]. In this context, negative self‐perception of the smile can be understood as the emotional outcome of dissatisfaction, influenced by social, cultural, psychological, and environmental factors [7–9].

Motivational factors play a critical role in determining and predicting patient satisfaction with treatment outcomes [10]. Therefore, it is essential to assess a patient’s motivation for seeking orthodontic care during the initial consultation and to identify their personal treatment goals [11]. The desire to enhance dental appearance is often reported as the primary motivating factor, followed by the intention to improve facial harmony [12]. To assist patients in visualizing potential changes to their smiles, Invisalign has developed a post treatment smile simulation tool. SmileView (SV) is an interactive application designed to offer prospective Invisalign patients a virtual preview of how their smile might appear after treatment. Users can upload a current smile photograph or take a real time image using a webcam, after which the tool applies computer based simulation to illustrate progressive dental alignment with Invisalign aligners. The software automatically detects the visible dentition, generates a modified image by applying standardized alignment models derived from typical Invisalign outcomes, and superimposes the simulated result onto the original smile photograph. It is important to note that SV provides only an esthetic projection of potential changes and does not constitute a personalized treatment plan.

Despite the increasing use of such tools, the esthetic self‐perception impact of smile simulation on individuals has not yet been systematically evaluated. It is, therefore, relevant to investigate the effect of this application on smile perception by analyzing specific esthetic variables. Finally, This study aims to assess whether using a smile simulator affects an individual’s motivation to pursue orthodontic treatment, to identify the smile features that most strongly contribute to this motivational change, to characterize the sociodemographic profile of individuals who are most receptive to the intervention, and to determine which esthetic changes perceived after viewing the SV simulation are associated with a greater intention to undergo aligner treatment.

## 2. Materials and Methods

### 2.1. Study Design

A prospective longitudinal study was conducted in two phases: T0 (baseline evaluation of smile characteristics using an initial photograph) and T1 (evaluation following smile modification using the SV application).

### 2.2. Study Population

A power analysis was performed a priori to ensure an adequate sample size for the primary outcome of smile perception variation. Based on a minimally important difference of 1.5 points on a 1–10 visual analog scale, and a standard deviation of 2.0 extracted from a previous study using similar esthetic criteria [13], a sample size of at least 16 participants was required to detect a statistically significant difference with 80% power at *α* = 0.05.

A total of 51 individuals were recruited in Madrid, Spain, from commercial centers, educational institutions, and office environments. Exclusion criteria included visible carious lesions in the smile area, missing teeth, periodontal disease, or any significant facial anomaly (Table 1).

**Table 1:** Participant demographics.

Variable	*N* (%)	Mean ± SD
Age (years)	—	37.4 ± 15.3
Sex		
Female	32 (62.7)	—
Male	19 (37.3)	—
Socioeconomic status		
Middle	35 (68.6)	—
High	13 (25.5)	—
Low	3 (5.9)	—
Education		
University	39 (76.5)	—
High school	7 (13.7)	—
No high school	5 (9.8)	—

### 2.3. Ethical Considerations

The study protocol was reviewed and approved by the Ethics Committee of Rey Juan Carlos University, Madrid (internal reference number: 2807202329823). The purpose of the study was explained to all participants, and the confidentiality of collected data was assured. Informed consent was obtained from all subjects before participation.

### 2.4. Procedure

The operator took a frontal photograph of each subject displaying a social smile (T0), following the specific instructions provided by the SV application. Participants then followed the steps to simulate their modified smile (T1) using the app, which gene‐rated a new image showing the projected post‐treatment appearance [14].

### 2.5. Instruments and Measurements

Following the simulation, participants received a questionnaire in Spanish (Table 2).

**Table 2:** Esthetic smile perception questionnaire.

Section 1	
1. Age	—
2. Gender	☐ Male ☐ Female ☐ Other
3. What is your socioeconomic status?	☐ Low ☐ Middle ☐ High
4. What is your educational level?	☐ Did not complete high school ☐ High school graduate ☐ University/higher education

Section 2 (view your selfie before it is modified by the application)	
1. Rate the overall esthetics of your smile (1 = i don’t like my smile at all, 10 = my smile is perfect)	☐1 ☐2 ☐3 ☐4 ☐5 ☐6 ☐7 ☐8 ☐9 ☐10
2. Rate the width of your smile (1 = very narrow, 10 = very wide)	☐1 ☐2 ☐3 ☐4 ☐5 ☐6 ☐7 ☐8 ☐9 ☐10
3. Rate the alignment of your teeth when smiling (1 = severely crowded, 10 = perfectly aligned)	☐1 ☐2 ☐3 ☐4 ☐5 ☐6 ☐7 ☐8 ☐9 ☐10
4. Rate the color of your teeth (1 = i hate the color, 10 = i really like it)	☐1 ☐2 ☐3 ☐4 ☐5 ☐6 ☐7 ☐8 ☐9 ☐10
5. Rate your gum exposure when smiling (1 = gummy smile, 10 = perfect gum display)	☐1 ☐2 ☐3 ☐4 ☐5 ☐6 ☐7 ☐8 ☐9 ☐10
6. Rate the shape of your teeth (1 = unattractive, 10 = very esthetic)	☐1 ☐2 ☐3 ☐4 ☐5 ☐6 ☐7 ☐8 ☐9 ☐10
7. Would you like to undergo orthodontic treatment?	☐ Yes ☐ No

Section 3 (view your selfie after modification with the application)	
1. Rate the overall esthetics of your smile (1 = i don’t like my smile at all, 10 = my smile is perfect)	☐1 ☐2 ☐3 ☐4 ☐5 ☐6 ☐7 ☐8 ☐9 ☐10
2. Rate the width of your smile (1 = very narrow, 10 = very wide)	☐1 ☐2 ☐3 ☐4 ☐5 ☐6 ☐7 ☐8 ☐9 ☐10
3. Rate the alignment of your teeth when smiling (1 = severely crowded, 10 = perfectly aligned)	☐1 ☐2 ☐3 ☐4 ☐5 ☐6 ☐7 ☐8 ☐9 ☐10
4. Rate the color of your teeth (1 = i hate the color, 10 = i really like it)	☐1 ☐2 ☐3 ☐4 ☐5 ☐6 ☐7 ☐8 ☐9 ☐10
5. Rate your gum exposure when smiling (1 = gummy smile, 10 = perfect gum display)	☐1 ☐2 ☐3 ☐4 ☐5 ☐6 ☐7 ☐8 ☐9 ☐10
6. Rate the shape of your teeth (1 = unattractive, 10 = very esthetic)	☐1 ☐2 ☐3 ☐4 ☐5 ☐6 ☐7 ☐8 ☐9 ☐10
7. Would you like to undergo orthodontic treatment?	☐ Yes ☐ No

In the first section of the questionnaire, participants were asked to evaluate their initial (T0) smile by rating the following variables on a scale from 1 to 10: overall smile perception, smile width, dental alignment, tooth color, gingival display, and tooth shape during smiling. They were then asked whether they would consider undergoing orthodontic treatment.

In the second section, based on the simulated image (T1), participants were asked to reevaluate the same variables using the same scale. Finally, they were asked again whether they would consider undergoing orthodontic treatment based on the potential outcome shown in the simulation.

### 2.6. Statistical Analysis

Data collected were analyzed using SPSS version 28.0 (IBM Corp., Armonk, NY, USA). For comparisons between T0 and T1 on continuous variables rated from 1 to 10 (e.g., general smile perception, alignment, color, shape, gingival exposure, and smile width), the paired Student’s *t*‐test was applied. For categorical dichotomous variables (e.g., willingness to undergo orthodontic treatment), the chi‐square test was used. To analyze the predictors of intention to undergo treatment with aligners after viewing the SV simulator, binary logistic regression was used. The dependent variable was intention to undergo treatment with aligners (coded as 0 = no intention, 1 = intention). The independent variables included perceived changes (*Δ*) in five esthetic dimensions comparing before and after viewing SV: smile width, tooth alignment, tooth color, gingival exposure when smiling, and tooth shape. All independent variables were measured as perceived differences (T1 − T0). The analysis was performed with a significance level of *p*  < 0.05. The *B* coefficients, standard errors, Wald statistics, *p*‐values, odds ratios (Exp[B]), and 95% confidence intervals were reported for each predictor. The significance level was set at *α* ≤0.05.

### 2.7. Bias

Potential subjectivity bias was acknowledged. All evaluations were self‐reported. No blinding was applied due to the visual nature of the intervention.

## 3. Results

All 51 recruited participants completed both T0 and T1 assessments. No dropouts or exclusions occurred after enrollment. General smile perception significantly increased after the SV simulation, rising from 5.84 ± 2.12 at T0 to 7.00 ± 2.61 at T1 (*p* = 0.038). Tooth alignment showed the most substantial improvement, from 5.63 ± 2.57 to 8.57 ± 1.67 (*p*  < 0.001). Significant changes were also observed for tooth color (from 5.31 ± 2.12 to 6.67 ± 2.76, *p* = 0.004) and tooth shape (from 6.18 ± 2.12 to 7.49 ± 2.52, *p* = 0.008). In contrast, changes in smile width (*p* = 0.143) and gingival display (*p* = 0.721) were not statistically significant.

The results indicate that, among subjects with a lower general perception of their smile after the SV simulation, the variable related to tooth shape showed a significant decrease, from 7.85 ± 1.62 at T0 to 5.38 ± 3.17 at T1 (*p* = 0.033). Smile width perception also decreased, although not significantly, from 7.46 ± 1.71 at T0 to 5.77 ± 2.92 at T1 (*p* = 0.059). Similarly, nonsignificant decreases were observed in tooth color perception (6.15 ± 1.99 at T0 vs. 5.46 ± 3.25 at T1, *p* = 0.528) and gingival exposure perception (7.92 ± 2.25 at T0 vs. 6.77 ± 2.86 at T1, *p* = 0.141). Tooth alignment perception was the only variable that showed a slight increase, from 7.08 ± 2.81 at T0 to 7.54 ± 2.47 at T1, although this change was not statistically significant (*p* = 0.674).

Following the SV simulation, participants’ willingness to undergo orthodontic treatment shifted slightly. At T0, 25 subjects (49.0%) expressed an intention to proceed with treatment, but this number declined to 23 (45.0%) at T1. Among those initially willing (T0), 15 (29.4%) maintained their decision, while 10 (19.6%) reversed it. Conversely, of the 26 participants who were initially unwilling, 8 (15.7%) changed their minds after the simulation, whereas 18 (35.3%) continued to reject the idea of treatment.

Key variables influencing the decision to pursue orthodontic treatment after the SV simulation were identified, along with a profile of subjects particularly receptive to its effects. Tooth alignment perception showed a significant improvement, increasing from 6.50 ± 1.06 at T0 to 8.88 ± 0.83 at T1 (*p* = 0.002). A similar trend was observed for tooth color, which rose from 5.50 ± 0.92 to 7.63 ± 1.40 (*p* = 0.002), and tooth shape, which improved from 6.25 ± 1.16 to 9.00 ± 0.92 (*p* = 0.001). In contrast, no statistically significant changes were recorded for smile width (6.63 ± 1.84 at T0 vs. 7.38 ± 1.84 at T1, *p* = 0.170) or gingival exposure (7.00 ± 1.69 at T0 vs. 8.13 ± 1.24 at T1, *p* = 0.080). The profile of individuals influenced by the simulation was predominantly female (62.5%), with an average age of 41, a middle to high socioeconomic status, and a university‐level education.

As can be seen in Table 3, logistic regression was performed to predict intention to undergo treatment with aligners after viewing SV. The model was statistically significant (*χ* ^2^ = 16.24, *p* = 0.006), explaining 36.5% of the variance (Nagelkerke’s *R* ^2^) and correctly classifying 68.6% of cases. The only significant variable was the perceived improvement in smile width (*p* = 0.039), with an odds ratio of 1.69. This indicates that those who perceived an improvement in their smile width were more likely to express an intention to undergo treatment with aligners. None of the other variables (alignment, color, gingival exposure, and tooth shape) showed a significant contribution to the model.

**Table 3:** Changes in willingness to undergo orthodontic treatment before (T0) and after (T1) smile simulation.

Would you undergo an orthodontic treatment?	Yes (T1)	No (T1)	Total	*X* ^2^ (*p*‐Value)
Yes (T0)	15 (29.4%)	10 (19.6%)	25 (49.0%)	4.398 (0.036)
No (T0)	8 (15.7%)	18 (35.3%)	26 (50.9%)	
Total	23 (45.0%)	28 (54.9%)	—	

*Note: X* ^2^, chi‐square test. Yes, indicates the willingness to undergo orthodontic treatment. No, indicates the refusal to undergo treatment.

## 4. Discussion

The results show a significant improvement in general smile perception after the SV simulation. This indicates an enhancement in self‐perception among the subjects studied. Smile esthetics are a key motivating factor for undergoing orthodontic treatment. Young patients often associate improved dental appearance with social and emotional benefits, such as higher self‐esteem and greater social acceptance [15, 16]. Moreover, dental imperfections, especially when highlighted by their correction during the simulation and often perceived as “anomalies,” create a strong desire for normality and attractiveness, reinforcing the motivation to pursue treatment [17].

First, the analysis of smile width showed no significant perceived change after the SV simulation. These findings do not align with what is commonly reported in the literature. Increased smile width generally has a positive influence on perceived attractiveness, being viewed more favorably when it is wider [18]. This discrepancy may be due to the noticeable perceived changes in other factors, such as alignment or tooth color, which are particularly prominent for the general public [19, 20]. These more visible features might have overshadowed the impact of smile width on overall smile perception.

Tooth alignment, on the other hand, had a significant effect on esthetic perception. Misalignment or spacing between teeth is often easily noticed by laypeople and plays a key role in evaluating smile attractiveness [19, 21]. These results suggest that dental alignment is a central concern for potential new patients. Thus, orthodontists should prioritize this factor when presenting treatment goals to help motivate patients.

The study also found a significant improvement in tooth color perception after using SV. This reflects the importance patients place on tooth color when assessing their smile’s appearance. One study identified tooth color as the leading cause of esthetic dissatisfaction among patients. An inappropriate shade can negatively impact overall smile perception and encourage individuals to seek corrective treatments. This highlights the critical role of tooth color in self‐perception and its influence on the decision to begin esthetic treatment [21, 22]. It is important to note, however, that orthodontic treatment with aligners does not directly improve tooth color. On the contrary, teeth may appear more yellow during treatment due to prolonged aligner use, especially if oral hygiene recommendations are not followed [23]. Therefore, SV capitalizes on patients’ high sensitivity to tooth color, which could lead to dissatisfaction at the end of treatment. This should be communicated to manage patient expectations.

Regarding gingival exposure, no significant improvement was reported following the simulation. Research shows that gingival exposure has a limited impact on esthetic perception among laypeople. These criteria tend to matter more to orthodontists than to patients, who focus more on visible elements like alignment or tooth proportions [5, 6]. While gingival exposure does contribute to smile esthetics, its influence depends on the degree of exposure [24, 25]. Limited exposure (less than 3 mm) is generally perceived as harmonious and esthetic, whereas excessive exposure is often viewed as less attractive. Consequently, a noticeable improvement would be more expected in individuals with over 3 mm of gingival display, which may explain the lack of significance in a general population sample.

The simulation also led to a significant improvement in the perception of tooth shape, The concept of tooth shape can be interpreted in several ways by the public. It may refer to tooth size and the proportions between central incisors, lateral incisors, and canines. These ratios play a critical role in the esthetic balance of a smile [26, 27]. In this context, the orthodontist plays a fundamental role. For example, interproximal enamel reduction (stripping) can be used to restore ideal inter‐ and intra‐arch proportions (Bolton ratio) during treatment [28]. Additionally, tooth shape may relate to anatomical features, including wear, microfractures, or irregularities in crown contours. These elements fall more within the scope of restorative dentistry than orthodontics. As with tooth color, the perceived improvements in these aspects during the simulation are illusory and may require further dental interventions, increasing overall treatment costs.

**Figure 1:**
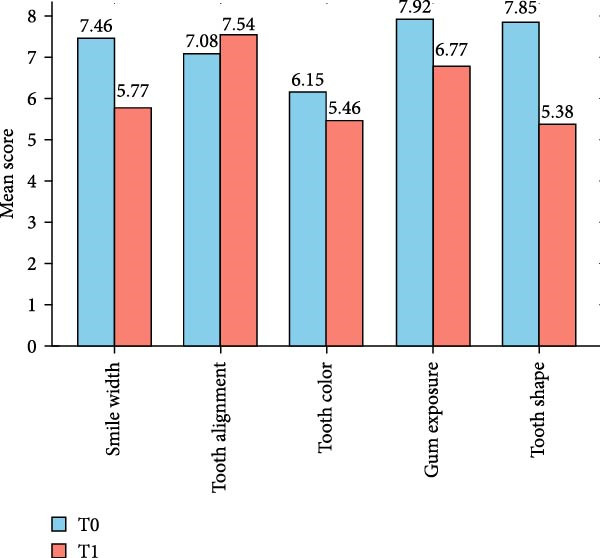
Analysis of smile related variables in patients with lower general smile perception scores at T1.

It is important to highlight the subjectivity of esthetic impact in malocclusions, which can be intensified by psychosocial factors, such as self‐esteem and individual esthetic perception [29, 30]. In this context, the T0 variable averages shown in Table 4 are consistently higher than those in Figure 1. This suggests that individuals whose general smile perception decreased between T0 and T1 already had a very positive self‐evaluation across all dimensions. This points to the influence of psychological factors like high self‐esteem and strong self‐confidence. A 2016 study found that individuals with high self‐esteem perceived less need for orthodontic treatment even when malocclusion was clinically diagnosed. The perception of treatment necessity is therefore strongly influenced by self‐esteem and personal esthetic standards [31]. These findings help explain the reduction in variable scores observed at T1.

**Table 4:** Impact of SV simulation on esthetic smile perception.

Item of esthetic smile perception	Mean	Standard deviation	*p*‐Value
Overall smile perception T0	5.84	2.12	0.038
Overall smile perception T1	7.00	2.61	
Smile width perception T0	6.59	2.13	0.143
Smile width perception T1	7.08	2.09	
Tooth alignment perception T0	5.63	2.57	<0.001
Tooth alignment perception T1	8.57	1.67	
Tooth color perception T0	5.31	2.12	0.004
Tooth color perception T1	6.67	2.76	
Gum exposure perception T0	7.80	2.23	0.721
Gum exposure perception T1	7.71	2.50	
Tooth shape perception T0	6.18	2.12	0.008
Tooth shape perception T1	7.49	2.52	

Interestingly, tooth alignment was the only variable that increased at T1 in these subjects, although the change was not statistically significant. This aligns with previous observations highlighting the high value the public places on alignment in smile esthetics. Well aligned teeth have been shown to positively influence social and cultural perceptions, affecting attributes, such as intelligence, social status, and attractiveness [2, 32]. This underlines the specific importance of alignment for the individuals in this subgroup.

In contrast to patients who experienced a decrease in general smile perception, those who reported an improvement had very low initial scores across all assessed characteristics. Significant and statistically relevant differences between T0 and T1 were observed for all variables except gingival exposure. This pattern reinforces earlier observations that gingival exposure is not a major concern for the general public, especially when it is under 3 mm during smiling.

A study on Brazilian adolescents found that low smile‐related self‐esteem was often linked to anterior crowding greater than 2 mm (reflected in the “alignment” variable) and diastemas over 2 mm (reflected in either “alignment” or “shape”) [33]. These two variables were particularly low at T0, indicating poor self‐esteem and low self‐confidence in these individuals. Such low initial values may be explained by a negative feedback loop in the self‐esteem cycle. Adolescents with a negative self‐image tend to assign disproportionate importance to moderate or barely visible malocclusions [34].

In summary, tooth alignment, color, and shape are the main features that individuals with low self‐esteem, and thus potential orthodontic patients, aim to improve. During initial consultations, patients are not always able to clearly express the nature of their esthetic concerns. These findings highlight the importance of practitioners explaining the limitations and possibilities of orthodontic treatment early on to properly manage patient expectations.

Findings regarding patients’ willingness to undergo treatment after the SV simulation were mixed. Although the simulation convinced 30.7% of initially hesitant participants at T0 to consider treatment (conversion rate), this must be weighed against the losses observed among those who initially favored treatment but changed their minds after seeing the simulated outcome. This group represents 40.0% of those with a favorable initial opinion. Overall, the number of subjects willing to undergo orthodontic treatment decreased from T0 to T1 (Table 3).

It is worth noting that SV was used outside the context of a formal orthodontic consultation. As a result, participants were exposed to a raw simulation image without professional explanation or guidance on how to interpret the results. This lack of context may have influenced their perception and final decision.

Among the subjects convinced of the need for orthodontic treatment, a typical profile emerged. These individuals were primarily women (62.5%) with an average age of 41, a middle to high socioeconomic status, and a university level education. This aligns with studies indicating that adult women are particularly motivated to improve their oral health, representing 72% of treatment initiations [35, 36]. Other research has shown that women, young adults, and those with prior orthodontic treatment tend to be more dissatisfied with their dental esthetics and more likely to seek care [37]. However, it is important to distinguish between the intention to consult an orthodontist and the sensitivity to SV simulations. Patients seeking care due to orthodontic relapse tend to be more demanding, more knowledgeable, and more critical. This type of patient may also be more responsive to digital tools.

As for age, active young adults are generally more attracted to digital interfaces, making them more capable of using and interpreting them [38]. This may explain their more nuanced reactions to the simulation.

The analysis of esthetic factors associated with changes in treatment decisions reveals distinct perceptual dynamics. Among patients who were initially unconvinced, certain dimensions such as tooth alignment, color, and shape appear to play a notable role in how they reevaluate their smile and develop an interest in treatment (Table 5). These features, traditionally linked to dental esthetics, may act as cognitive triggers, activating a latent desire for transformation. However, when considering the overall effect of the simulation on treatment intention, a different interpretation emerges. Some esthetic changes, while positively perceived, do not consistently translate into a willingness to proceed with treatment. In contrast, the perceived increase in smile width seems to function as a particularly powerful emotional trigger likely due to its immediate visual impact and its association with socially valued traits (Table 6). This contrast highlights the potential gap between esthetic appreciation and actual behavioral motivation. Nevertheless, this interpretation should be approached with caution. Some participants already expressed a favorable treatment intention before viewing the simulation. Their final decision, therefore, cannot be attributed solely to the esthetic changes perceived.

**Table 5:** Analysis of smile related variables influencing subjects’ decisions to undergo orthodontic treatment.

Items of esthetic smile perception	Mean	Standard deviation	*p*‐Value
Smile width perception T0	6.63	1.84	0.170
Smile width perception T1	7.38	1.84	
Tooth alignment perception T0	6.50	1.06	0.002
Tooth alignment perception T1	8.88	0.83	
Tooth color perception T0	5.50	0.92	0.002
Tooth color perception T1	7.63	1.40	
Gum exposure perception T0	7.00	1.69	0.080
Gum exposure perception T1	8.13	1.24	
Tooth shape perception T0	6.25	1.16	0.001
Tooth shape perception T1	9.00	0.92	

**Table 6:** Logistic regression results for predicting the intention to undergo treatment with aligners after viewing SmileView (*N* = 51).

Predictor	B	SE	Wald	df	*p*‐Value	OR (Exp[B])	95% CI for OR
*Δ* Smile width	0.524	0.255	4.245	1	0.039 ^∗^	1.69	[1.026, 2.783]
*Δ* Tooth alignment	−0.034	0.158	0.047	1	0.829	0.966	[0.708, 1.318]
*Δ* Tooth color	0.098	0.149	0.43	1	0.512	1.103	[0.823, 1.478]
*Δ* Gum exposure	0.294	0.231	1.622	1	0.203	1.341	[0.854, 2.108]
*Δ* Tooth shape	0.092	0.151	0.373	1	0.541	1.097	[0.816, 1.474]
Constante	−0.837	0.572	2.147	1	0.143	0.433	—

*Note: Δ* (T1 − T0).

Abbreviations: CI, confidence interval; OR, odds ratio.

^∗^ *p*  < 0.05.

### 4.1. Practical Implications

SV can serve as a powerful visual tool during the initial consultation. It helps patients visualize potential treatment outcomes, particularly those with low self‐esteem who tend to be more sensitive to improvements in dental alignment, color, and shape. Orthodontists can use this tool to strengthen patient motivation, provided it is accompanied by clear explanations about the limitations of aligner treatments. Although SV improves the esthetic perception of the smile, it can also generate unrealistic expectations, especially regarding aspects, such as tooth color or shape. These aspects often require additional multidisciplinary treatments. It is, therefore, essential to inform patients about the simulated nature of the results and the possible additional costs involved.

The typical profile of a patient influenced by the simulation is a 41‐year‐old woman with a middle to high socioeconomic status and a university level education. This information allows for the development of targeted communication strategies to increase persuasive impact, particularly in cases involving alignment issues or interdental spaces.

SV also assists in assessing a patient’s willingness to undergo treatment. However, the decrease in intention observed in some participants after viewing the simulation suggests the need to present results within a clinical setting. It is important to explain how esthetic variables relate to the treatment objectives.

This tool may also be incorporated into educational programs to raise awareness of the importance of dental alignment in both esthetic and social perception. This could be especially effective among younger adults or those motivated by professional or social goals. Moreover, SV could be improved by incorporating psychological factors, such as initial self‐esteem. Presimulation questionnaires could be used to adapt the modifications to the individual’s expectations, thereby increasing both satisfaction and motivational effectiveness.

### 4.2. Study Limitations

The investigation into the esthetic self‐perception impact of the SV simulation on smile perception presents certain limitations that should be taken into account. The use of subjective questionnaires to evaluate smile perception introduces a potential bias, as responses depend on individual interpretation and may be influenced by uncontrolled emotional or contextual factors. Furthermore, although the sample size was adequate according to the power analysis, it was limited to 51 participants from Madrid. This may restrict the generalizability of the findings to populations with different cultural or socioeconomic backgrounds. However, findings may be generalizable to urban, educated, middle‐to‐high SES adult populations in developed countries. Additionally, the absence of a control group not exposed to SV makes it difficult to determine whether the observed changes in perception were solely due to the simulation or influenced by external factors. Finally, the context in which SV was applied, outside of formal orthodontic consultation, may have affected how participants interpreted the results, as they lacked professional guidance to understand the limitations of the simulation.

Additionally, the reliability of self‐reported evaluations was not assessed through repeated measures (test–retest reliability), which could have further validated the consistency of participants’ responses over time. This omission is due to the immediate sequential nature of the T0 and T1 assessments, conducted in a single session to avoid short‐term recall bias. Repeating evaluations after a time interval would have been problematic, as the memory of the simulated image (T1) could have influenced new T0 evaluations, compromising the validity of the initial measures.

## 5. Conclusion

1. The results show a significant improvement in the overall perception of the smile after the simulation. The variables of alignment, color, and tooth shape perception improved significantly.
2. The set of studied variables is responsible for the decrease in the general smile perception score, except for the variable related to dental alignment.
3. The set of studied variables is responsible for the improvement in the general smile perception score, except for the variable related to gingival exposure.
4. A decrease has been observed in the number of subjects willing to undergo orthodontic treatment after viewing the smile simulation.
5. The factors influencing the change in decision after viewing the simulation include dental alignment, tooth color, and tooth shape, although the perceived increase in smile width appears to be the strongest independent predictor of treatment intention.
6. The target profile identified is that of a middle aged woman, approximately 41 years old, with a middle to high socioeconomic status and a university level education.

## Conflicts of Interest

The authors declare no conflicts of interest.

## Funding

This research received no external funding.

## Data Availability

The data supporting the findings of this study are not publicly available due to legal restrictions.
